# Positive regulation of Rho GTPase activity by RhoGDIs as a result of their direct interaction with GAPs

**DOI:** 10.1186/s12918-015-0143-5

**Published:** 2015-01-28

**Authors:** Takahide Ota, Masayo Maeda, Mayumi Okamoto, Masaaki Tatsuka

**Affiliations:** Division of Tumor Biology, Department of Life Science, Medical Research Institute, Kanazawa Medical University, Uchinada, Ishikawa 920-0293 Japan; Department of Chemistry, Kanazawa Medical University, Uchinada, Ishikawa 920-0293 Japan; Department of Life Sciences, Life and Environmental Sciences, Prefectural University of Hiroshima, Nanatsuka, Shoubara, Hiroshima 727-0023 Japan

**Keywords:** RhoGDI, Rho GTPases, Ordinary differential equation, GAPs, GEFs

## Abstract

**Background:**

Rho GTPases function as molecular switches in many different signaling pathways and control a wide range of cellular processes. Rho GDP-dissociation inhibitors (RhoGDIs) regulate Rho GTPase signaling and can function as both negative and positive regulators. The role of RhoGDIs as negative regulators of Rho GTPase signaling has been extensively investigated; however, little is known about how RhoGDIs act as positive regulators. Furthermore, it is unclear how this opposing role of GDIs influences the Rho GTPase cycle. We constructed ordinary differential equation models of the Rho GTPase cycle in which RhoGDIs inhibit the regulatory activities of guanine nucleotide exchange factors (GEFs) and GTPase-activating proteins (GAPs) by interacting with them directly as well as by sequestering the Rho GTPases. Using this model, we analyzed the role of RhoGDIs in Rho GTPase signaling.

**Results:**

The model constructed in this study showed that the functions of GEFs and GAPs are integrated into Rho GTPase signaling through the interactions of these regulators with GDIs, and that the negative role of GDIs is to suppress the overall Rho activity by inhibiting GEFs. Furthermore, the positive role of GDIs is to sustain Rho activation by inhibiting GAPs under certain conditions. The interconversion between transient and sustained Rho activation occurs mainly through changes in the affinities of GDIs to GAPs and the concentrations of GAPs.

**Conclusions:**

RhoGDIs positively regulate Rho GTPase signaling primarily by interacting with GAPs and may participate in the switching between transient and sustained signals of the Rho GTPases. These findings enhance our understanding of the physiological roles of RhoGDIs and Rho GTPase signaling.

**Electronic supplementary material:**

The online version of this article (doi:10.1186/s12918-015-0143-5) contains supplementary material, which is available to authorized users.

## Background

Rho family GTPases are members of the Ras GTPase superfamily and act as molecular switches in numerous signaling pathways that control a variety of cellular processes, including actin cytoskeletal organization, microtubule dynamics, vesicle trafficking, cell cycle progression, and cell polarization [1]. Most Rho GTPases cycle between active GTP-bound and inactive GDP-bound states. There are three classes of regulators of Rho GTPases, namely, guanine nucleotide exchange factors (GEFs), GTPase-activating proteins (GAPs), and GDP-dissociation inhibitors (GDIs).

GEFs activate GTPases by promoting the exchange of GDP for GTP. GAPs inactivate GTPases by stimulating their intrinsic GTP-hydrolyzing activity. GDIs are known to regulate only members of the Rho and Rab families and not other families of the Ras superfamily, although a GDI-like protein for Ras GTPases has been reported [2]. Unlike GEFs and GAPs, GDIs play several roles in the regulation of the Rho family GTPases [3-6]. First, GDIs bind GDP-bound GTPases and inhibit the dissociation of GDP from GTPases, thereby preventing the promotion of GDP/GTP exchange by GEFs and maintaining the GTPases in an inactive state [7]. Second, although the binding affinity of GDIs to GTP-bound GTPases remains controversial [8-15], it is possible that GDIs bind GTP-bound GTPases and inhibit both intrinsic and GAP-promoted GTP hydrolyzing activity [8,16,17], thereby maintaining GTPases in an active state. Third, GDIs mediate the cycling of GTPases between cytosolic and target sites [7].

GDIs for the Rho family GTPases can therefore act to inhibit both the activation and inactivation of GTPases by interacting with GDP- and GTP-bound GTPases, respectively. This dual function of GDIs is noteworthy, and adds to our understanding of the regulatory mechanisms of the Rho GTPase cycle, because GDIs for Rab family GTPases show a marked preference for the GDP-bound form [18]. Furthermore, it has also been suggested that Rho GTPases are regulated by a fine balance between GEF and GAP activities, and that the inactivation of GAP activity is a physiologically important regulatory mechanism for activating Rho GTPases [19]. Nonetheless, little is known about the significance of the inhibition of GAP-promoted GTP hydrolyzing activity by GDIs in the regulation of Rho signaling. How the opposing roles of GDIs influence the Rho GTPase cycle is also unclear.

Several ordinary differential equation models and process models of the Rho GTPase cycle have been constructed and analyzed [20-24]. In these models, GDIs inhibit the functions of GEFs and GAPs by sequestering GDP-bound and GTP-bound GTPases, respectively. However, the actual mechanisms involved in GDI inhibition of GEF and GAP activity are not fully understood. A previous report suggested that RhoGDIs can physically interact directly with both GEFs [25] and GAPs [26]. Based on these observations, we constructed a model of the Rho GTPase cycle in which GDIs inhibit the activities of GEFs and GAPs not only by sequestering GTPases, but also by direct physical interaction.

Using this model, we showed that the functions of GEFs and GAPs are integrated into Rho GTPase signaling through the interactions of these regulators with GDIs and that the negative role of GDIs is to suppress the overall Rho activity by inhibiting GEFs. Additionally, the positive role of GDIs is to sustain Rho activation by inhibiting GAPs. These observations illustrate the more detailed roles RhoGDIs and further enhance our understanding of the physiological functions of Rho GTPase signaling.

## Results

### Interaction of GDI with GAP sustains Rho activation

**Figure 1:**
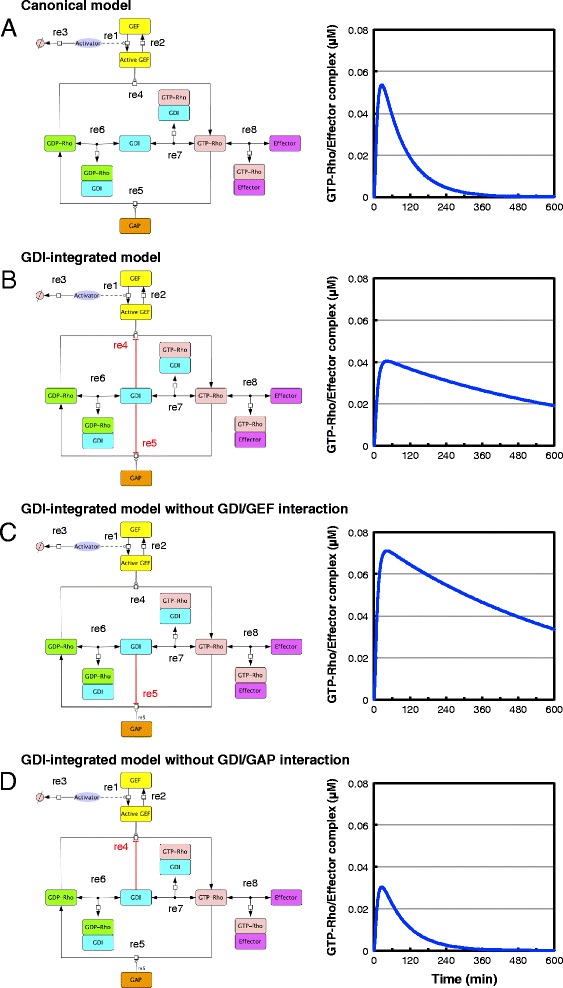
**Representation of the models of Rho GTPase cycle regulation (left) and simulations of their Rho activation dynamics (right).** The activation levels of GTPases were defined as the concentration of the GTP-Rho/Effector complex. **A)** The canonical model of the Rho GTPase cycle in which GDIs inhibit the activities of GEFs and GAPs by sequestering GTPase. **B)** The GDI-integrated model of the Rho GTPase cycle in which GDIs inhibit the activities of GEFs and GAPs not only by sequestering GTPase but also by interacting with GEFs and GAPs. **C)** GDI/GEF interaction was removed from the GDI-integrated model. **D)** GDI/GAP interaction was removed from the GDI-integrated model. All parameters and reactions in the models are shown in Additional file 1: Tables S1 and S2. Reaction numbers (re#) correspond to the reaction numbers in Additional file 1: Table S2.

A Rho GTPase switch can be regulated by three classes of regulators: GEFs, GAPs, and GDIs. In the canonical model of the Rho GTPase cycle (Figure 1A, left), GEFs promote GDP/GTP exchange, thereby activating Rho GTPases. In contrast, GAPs promote GTP hydrolysis, thereby inactivating Rho GTPases. GDIs sequester GDP-bound GTPases from GEFs and keep them inactive; however, GDIs can also sequester GTP-bound GTPases from GAPs and keep them active. In this model, the Rho GTPase cycle functions as a simple ON/OFF switch and Rho activation is transiently elevated upon stimulation (Figure 1A, right).

The majority of Rho GTPases exist in biologically inactive cytosolic complexes with GDIs, and the dissociation of GTPases from GDIs is hypothesized to be a prerequisite for activation by GEFs. However, it has been suggested that GDI and Rho GTPase can simultaneously bind GEF or GAP and form a ternary complex (GEF/GDI/Rho GTPase or GAP/GDI/Rho GTPase) [25-27]. According to these observations, we constructed a model of the Rho GTPase cycle (Figure 1B, left) in which GDIs inhibit the activities of GEFs and GAPs by physically interacting with them as well as by sequestering Rho GTPases (see Methods). We designated this model the ‘GDI-integrated model’ because the activation dynamics and ultimate output of GEFs and GAPs are integrated by GDIs to regulate Rho activity. Rho activation is sustained for a longer period of time in this model (Figure 1B, right), compared with the canonical model (Figure 1A, right).

To clarify which interaction of GDIs with GEFs or GAPs participates in this sustained Rho activation, we further modified our GDI-integrated model. When the interaction of GDIs with GEFs was removed (Figure 1C, left), similar Rho activation dynamics, with a two-fold increase in the overall level, were obtained (Figure 1C, right). In contrast, when the interaction of GDIs with GAPs was removed (Figure 1D, left), Rho activation level decreased and was not sustained (Figure 1D, right). These results therefore suggest that GDIs sustain Rho activation through interaction with GAPs.

### Influence of free (non-GTPase-complexed) GDI levels on Rho activation dynamics

**Figure 2:**
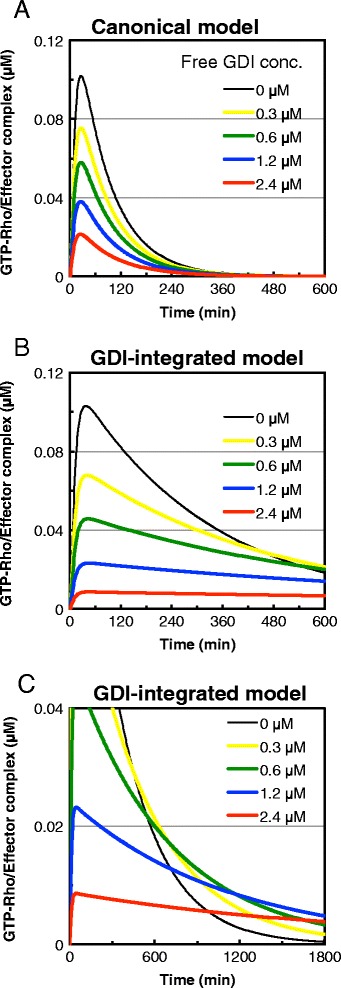
**Free (non-GTPase-complexed) GDI concentration affects the prolongation of Rho activation in the GDI-integrated model.** Rho activation dynamics were simulated at various concentration of free GDI. **A)** 600 min after stimulation in the canonical model. **B)** 600 min after stimulation in the GDI-integrated model. **C)** 1,800 min after stimulation in the GDI-integrated model. The activation levels of GTPases were expressed as the concentration of GTP-Rho/Effector complex.

To confirm the contribution of GDIs in sustaining Rho activation, we simulated Rho activation dynamics in the presence of various cellular concentrations of free GDIs, i.e., GDIs not complexed with GTPases. Based on the literature [28], we calculated the concentration of free RhoGDIα to be 0.7 μM (Additional file 1: Table S1). We used a range of concentrations of free GDIs close to this value to simulate the Rho activation dynamics. The canonical model predicted that an increase in free GDIs would simply lead to an overall decrease in Rho activation (Figure 2A). However, in our GDI-integrated model, while the increase of free GDIs also led to an overall decrease in Rho activation, this did not negate the sustained Rho activation (Figure 2B). Unexpectedly, the presence of free GDIs sustained the Rho activation level beyond 1,800 min after stimulation, in contrast to the cessation observed at this time point in the absence of free GDIs (Figure 2C).

### Influence of GDI affinity for GEF and GAP and the concentration of GEF and GAP on Rho activation dynamics

**Figure 3:**
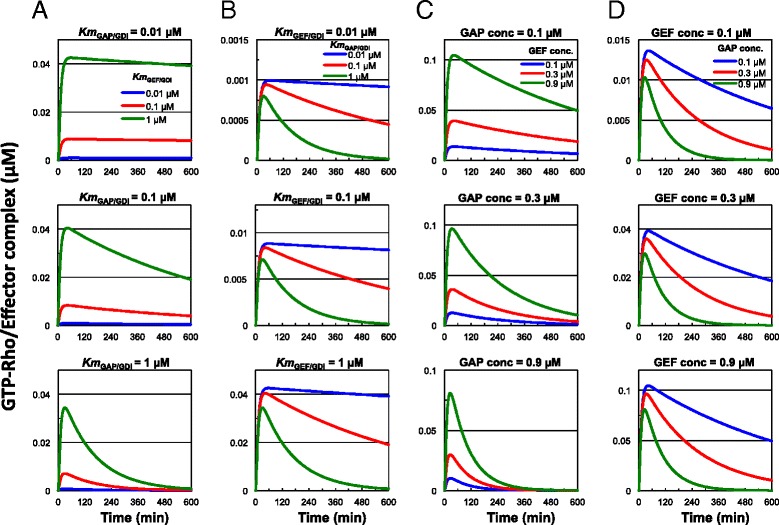
**Prolongation of Rho activation in the GDI-integrated model is dependent on** ***K***_***m*GAP/GDI**_ **and the GAP concentration.** Rho activation dynamics were simulated at various *K*_*m*GEF/GDI_ values **(A)**, *K*_*m*GAP/GDI_ values **(B)**, GEF concentration **(C)**, and GAP concentrations **(D)** in the GDI-integrated model. The activation levels of GTPases were expressed as the concentration of GTP-Rho/Effector complex.

Phosphorylation affects the affinity of GDIs for various Rho GTPases [29-33] and affects the function of GEFs [34,35] and GAPs [36-38]. Therefore, phosphorylation may modify the regulation of Rho signaling by GDIs, GEFs and GAPs. To examine how the affinity of GDIs for GEFs (*K*_*m*GEF/GDI_) and GAPs (*K*_*m*GAP/GDI_) affects the ability of GDIs to sustain Rho activation, we simulated the Rho activation dynamics at 0.01, 0.1, and 1.0 μM of *K*_*m*GEF/GDI_ and *K*_*m*GAP/GDI_ in our model. The decrease of *K*_*m*GEF/GDI_ resulted in overall decrease of Rho activation at all the tested concentrations of *K*_*m*GAP/GDI_ (Figure 3A). The Rho activation was markedly sustained at 0.01 and 0.1 μM of *K*_*m*GAP/GDI_ and decreasing *K*_*m*GEF/GDI_ did not negate the sustained Rho activation (Figure 3A). Conversely, as the *K*_*m*GAP/GDI_ value became smaller, the Rho activation was sustained to a greater degree at all *K*_*m*GEF/GDI_ (Figure 3B). These results indicate that the sustained Rho activation can primarily be attributed to the interaction between GAPs and GDIs, and the higher affinity of GDIs for GAPs promotes sustained Rho activation.

**Figure 4:**
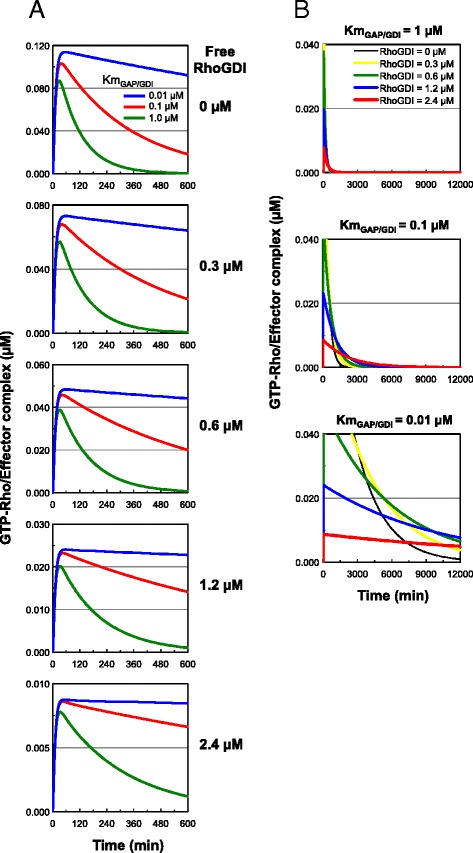
**GDI enables extremely long-term retention of the activation state of Rho GTPases.** Simulation of Rho activation dynamics at *K*_*m*GAP/GDI_ = 0.01, 01, and 1.0 μM in the presence of various free GDI concentrations (0–2.4 μM) in the GDI-integrated model. **A)** 600 min after stimulation. **B)** 12,000 min after stimulation. The activation levels of GTPases were expressed as the concentration of GTP-Rho/Effector complex.

It was also suggested that the local concentration of GEFs and GAPs defined the modes of Rho GTPase signaling [22]. We examined how the concentration of GEFs and GAPs affected the ability of GDIs to sustain Rho activation. We simulated the Rho activation dynamics at 0.1, 0.3, and 0.9 μM concentrations of GEFs and GAPs in our model. The decrease of GEF concentration resulted in overall decrease of Rho activation at all of the tested GAP concentrations (Figure 3C). The sustained Rho activation was apparent only at 0.1 μM of GAP and the decrease of GEF concentration did not negate this sustained Rho activation (Figure 3C). However, at all of the tested GEF concentrations, as the GAP concentrations became smaller, Rho activation was sustained to a higher degree, and increasing GAP concentration negated this sustained Rho activation (Figure 3D). These results indicate that the sustained Rho activation is dependent on the concentration of GAPs, and a lower GAP concentration sustains Rho activation. Finally, we compared the Rho activation dynamics at 0.01, 0.1, and 1.0 μM *K*_*m*GAP/GDI_ under various concentrations of free GDI. A decrease in the *K*_*m*GAP/GDI_ value enhanced the prolongation of Rho activation regardless of free GDI concentration (Figure 4A). Surprisingly, at 0.01 μM *K*_*m*GAP/GDI_, Rho activation was sustained for a significant period of time, longer than 12,000 min (8.3 days), after stimulation in the presence of free GDI (Figure 4B). However, the overall levels of Rho activation markedly decreased in association with an increase in free GDI. These results suggest that GDIs enable extremely long-term retention of the activated state of the Rho GTPases.

## Discussion

It is well established that the main function of RhoGDIs is to maintain Rho GTPases in inactive soluble complexes. In many canonical models of the Rho GTPase cycle, GDIs extract GTPases from the membrane and sequester them as inactive cytosolic complexes. RhoGDIs are therefore predominantly thought to act as negative regulators; however, they inhibit both activation [7] and inactivation [8,16,17] of GTPases. Little is known about how the opposing functions of GDIs influence the Rho GTPase cycle.

In the present study, we constructed a model of the Rho GTPase cycle, designated as the GDI-integrated model, in which GDIs inhibit the activities of GEFs and GAPs by interacting with them in addition to sequestering the Rho GTPases. This model indicated that GDIs sustain the activation of Rho GTPase by interacting with GAPs. Furthermore, as expected from the positive regulatory role of GDIs, (in other words, the inhibition of GAP activity by GDIs), an increase in the intracellular concentration of free GDIs enhanced the prolongation of Rho activation despite the overall decrease in the Rho activation level.

It was previously reported that the molar amount of RhoGDIα is roughly equal to the molar total of the RhoA, Rac1, and Cdc42 GTPases in several types of cultured cells [28]. RhoGDIβ is strongly expressed in hematopoietic cell lineages [39,40] and in other cell types [41-43], though it is not as ubiquitous as RhoGDIα. Specificities of RhoGDIs for Rho GTPases are largely unknown, but both RhoGDIα and RhoGDIβ can associate with RhoA, Cdc42, and Rac1 in some cell types [3]. Therefore, in RhoGDIβ-expressing cells, the total amount of RhoGDIα and RhoGDIβ may exceed the total amount of these Rho GTPases. In such cases, RhoGDIs may exist in a non-GTPase-complexed form in the cell and may function to sustain Rho activation for long periods.

RhoGDIβ is implicated in cancer progression, however, reports have presented contradictory evidence as to the nature of the correlation between cancer progression and RhoGDIβ expression level [44]. We have also reported that RhoGDIβ plays a positive [41,45,46] and negative [47] role in cancer progression. Several explanations for this contradictory behavior of RhoGDIβ have been proposed [44]. Our present study suggests that RhoGDI can act both as a positive and negative regulator of GTPases, and which role RhoGDI plays may depend on its expression level. This presents at least a partial explanation for the inconsistent correlation of RhoGDIβ with cancer progression.

It has been proposed that intracellular signals are transmitted through the dynamic activities of signaling molecules (defined as the temporal change in activity of a molecule) [48]. For example, in the case of ERK (extracellular signal-regulated kinases), transient and sustained activation states have been shown to result in different cellular responses [49]. It is well established that GEFs and GAPs function as positive and negative regulators of Rho GTPase cycles, respectively. We have shown that the functions of GEFs and GAPs are modulated by their interactions with GDIs, and that the interconversion between transient and sustained Rho activation occurs mainly through changes in the affinities of GDIs to GAPs and the concentrations of GAPs. The properties of GDIs and GAPs are regulated by posttranscriptional modifications [29-33,36-38] and the affinity between GDIs and GAPs may be altered by such modifications. Therefore, RhoGDIs and GAPs might participate in the switching between transient and sustained signals of the Rho GTPases. Although this mode seems not to be common in the regulation of Rho GTPases, certain sets of GTPases, GEFs, and GAPs may use this mode of regulation.

In the present study, we proposed a simplified model for positive regulation of Rho GTPases by GDIs. However, the model does not take into account GTPase cycling between membrane and cytosol. Cells contain membranous and cytoplasmic compartments, and typically, Rho GTPases function within the membranous compartments. It has been shown that the efficient cycling between inactive and active states of GTPases can occur entirely within protein complexes assembled on membrane surfaces [22]. RhoGDIs mediate the membrane-cytoplasmic shuttling of GTPases, and likely can alter the concentrations of GTPases and their RhoGDI-associated regulators at target sites in cells. Therefore, it is necessary to take into account the shuttling processes in developing a truly comprehensive model. Membrane-cytoplasmic shuttling has been considered in a simulation of the distribution of activated Cdc42 during the early phase of yeast bud formation [23]. Additionally, a modeling framework describing Rac cycling between membrane and cytosol has been reported [21]. Because our model for the Rho GTPase switch can be regarded as a basal signaling module, these studies that have taken into account the Rho GTPase shuttling processes should be incorporated into our model of the Rho GTPase switch for a more detailed and biologically-relevant model.

## Conclusions

We constructed models of the Rho GTPase cycle in which RhoGDIs inhibit the activities of GEFs and GAPs by physically interacting with them, as well as by sequestering the Rho GTPases. This model showed that the functions of GEFs and GAPs are integrated into Rho GTPase signaling through the interactions of these regulators with GDIs, and thus, the interconversion between transient and sustained Rho activation occurs by changes mainly in the affinities of GDIs to GAPs and the concentrations of GAPs. These results provide new insights into the physiological roles of Rho GTPase signaling.

## Methods

### Construction of models

The pathway diagrams of the Rho GTPase cycle and their simulation programs were described using CellDesigner (Systems Biology Institute, Tokyo, Japan) [50], and were simulated by SOSLib in CellDesigner. All kinetic reactions in the pathway diagrams in Figure 1 were described by ordinary differential equations based on mass-action kinetics (reactions 1, 2, 3, 6, 7, and 8) or Michaelis–Menten kinetics (reactions 4 and 5) [51,52].

In the canonical model (Figure 1A), we used a typical Michaelis–Menten kinetic model to describe the promoting activities of GEFs (reaction 4 in Figure 1A and C) and GAPs (reaction 5 in Figure 1A and D) towards the Rho GTPases. GDIs inhibit the activities of GEFs and GAPs only by sequestering Rho GTPases.

The majority of Rho GTPases exist in biologically inactive cytosolic complexes with GDIs and the dissociation of GTPases from GDIs is hypothesized to be a prerequisite for activation by GEFs. However, it has been suggested that βPIX (GEF), Rac1, and RhoGDIα form a ternary complex [27] and that Bcr (GAP), Rac, and RhoGDIα also form a ternary complex [26]. Furthermore, several studies have shown that GDIs directly interact with both GEFs [25] and GAPs [26]. These observations suggest that GDIs and Rho GTPases can simultaneously bind GEFs or GAPs, and form ternary complexes. According to these observations, we constructed a model of the Rho GTPase cycle (Figure 1B, left) in which GDIs inhibit the activities of GEFs and GAPs by interacting with them as well as by sequestering Rho GTPases. We used the non-competitive inhibition model of Michaelis–Menten kinetics to describe the reactions in which GDIs inhibit the actions of GEFs (reaction 4 in Figure 1B and D) and GAPs (reaction 5 in Figure 1B and C), because in the non-competitive inhibition model the inhibitor and substrate can simultaneously bind the enzyme. The processes of GTPase cycling between membrane and cytosol are very important for understanding Rho activation dynamics. However, in the present study we focused on the interaction of GDIs and GEFs/GAPs and address how GDIs regulate GTPase activity through these interactions. Therefore, to simplify the model we did not consider the membrane localization of Rho GTPases and their regulators in our models.

Parameters and equations in the models are listed in Tables S1 and S2 (Additional file 1). The kinetic parameters and initial concentrations of molecules were determined based on previous studies [24,28,53-56] or arbitrary values. The activation levels of GTPases were defined as the concentrations of the GTP-Rho/Effector complex. Model files are provided as .xml files (Additional files 2 and 3) in the supplementary materials and can be viewed using CellDesigner [50].

## Additional files

Additional file 1:
**Tables S1-2.pdf.** **Table S1.** lists the initial concentrations of molecules. **Table S2.** lists the kinetic reactions, ordinary differential equations, and parameters used in the models. Additional file 2:
**Canonical model.xml.** The canonical model file is provided as an .xml file and can be viewed using CellDesigner. Additional file 3:
**GDI-integrated model.xml.** The GDI-integrated model file is provided as an .xml file and can be viewed using CellDesigner.
