# Supplementary material for: Positive regulation of Rho GTPase activity by RhoGDIs as a result of their direct interaction with GAPs
Source: BMC Syst Biol. 2015 Jan 28;9:3. doi: 10.1186/s12918-015-0143-5 (PMC4312443; doi:10.1186/s12918-015-0143-5)
Supplement: Additional file 1: — Tables S1-2.pdf. Table S1. lists the initial concentrations of molecules. Table S2. lists the kinetic reactions, ordinary differential equations, and parameters used in the models. [file 12918_2015_143_MOESM1_ESM.pdf]

Table S1. Initial concentrations of molecules in a cell

Molecules	cellular concentration	References/Remarks
Activator	1 μM	arbitrary
GEF	0.31 μM	[Aoki, 2007]
Active GEF	0 μM	
GDP-Rho*	0 μM	
GTP-Rho*	0 μM	
free GDI**	0.7 μM	[Michaelson, 2001]
GDI・GDP-Rho***	1.3 μM	[Michaelson, 2001]
GDI・GTP-Rho***	0 μM	
GAP	0.1 μM	arbitrary
Effector	1 μM	arbitrary
GTP-Rho・Effector	0 μM	

Aoki K, Nakamura T, Inoue T, Meyer T, Matsuda M (2007) J Cell Biol 177: 817-827.
Michaelson D, Silletti J, Murphy G, D'Eustachio P, Rush M, Philips MR (2001) J Cell Biol 152:111-126.
Chuang TH, Bohl BP, Bokoch GM (1993) J Biol Chem 268:26206-26211.

* The molar amount of RhoGDI is roughly equal to the total levels of the RhoA, Rac1 and Cdc42 GTPases in several types of cultured cell [Michaelson, 2001] and the majority of GDP-bound Rac in cells is present in a complex with RhoGDI [Chuang, 1993], therefore, we estimated that the initial concentrations of GDP- and GTP-Rho that are not bound GDI are 0.

** Michaelson et al. [Michaelson, 2001] reported the cellular concentration of each molecule as follows,

RhoGDIα = 283 ± 27 ng/10^6 cells

RhoA = 56 ± 14 ng/10^6 cells

Rac1 = 124 ± 27 ng/10^6 cells

Cdc42 = 53 ± 18 ng/10^6 cells

Assuming cellular volume as 2 pl, we calculated the molar concentration of these molecules as follows,

RhoGDIα = 6.1 μM
RhoA = 1.3 μM
Rac1 = 2.9 μM
Cdc42 = 1.2 μM

We assumed that RhoA, Rac1, and Cdc42 were entirely complexed with RhoGDIα and calculated the concentration of free RhoGDIα as follows,

6.1 -(1.3 + 2.9 + 1.2) = 0.7 μM

***To analyze one species of Rho GTPase we choosed RhoA as an example. Therefore, we assumed the concentration of Rho GTPase as 1.3 μM. We also assumed that all RhoA was GDP-bound form before stimulation, therefore, the initial concentration of GDI/GDP-Rho and GDI/GTP-Rho complex was estimated as 1.3 and 0 μM, respectively.

Table S2. Kinetic reactions, equations, and parameteres in the models

Reaction number*	Models**	Reactions	Equations	Parameters	
				Values	References/Remarks
re1	A, B, C, D	$[\text{GEF}] + [\text{Activator}] \xrightarrow{k_1} [\text{Active GEF}]$	$\frac{d[\text{Active GEF}]}{dt} = k_1[\text{GEF}][\text{Activator}]$	$k_1 = 1\ \mu M^{-1} min^{-1}$	arbitrary
re2	A, B, C, D	$[\text{Active GEF}] \xrightarrow{k_2} [\text{GEF}]$	$\frac{d[\text{Active GEF}]}{dt} = -k_2[\text{Active GEF}]$	$k_2 = 0.1\ min^{-1}$	arbitrary
re3	A, B, C, D	$[\text{Activator}] \xrightarrow{k_3} [\text{degrade Activator}]$	$\frac{d[\text{Activator}]}{dt} = -k_3[\text{Activator}]$	$k_3 = 0.5\ min^{-1}$	arbitrary
re4	A, C	$[\text{GDP-Rho}] + [\text{Active GEF}] \overset{Km_{GEF/Rho}}{\rightleftarrows} [\text{GDP-Rho·Active GEF}] \xrightarrow{kcat_{GEF}} [\text{GTP-Rho}] + [\text{Active GEF}]$	$\frac{d[\text{GTP-Rho}]}{dt} = \frac{kcat_{GEF}[\text{GDP-Rho}][\text{Active GEF}]}{Km_{GEF/Rho} + [\text{GDP-Rho}]}$	$Km_{GEF/Rho} = 24.5\ \mu M$<br>$kcat_{GEF} = 5.64\ min^{-1}$	[Zhang, 2000]<br>[Zhang, 2000]
	B, D	$[\text{Active GEF}] + [\text{GDP-Rho}] \overset{Km_{GEF/Rho}}{\rightleftarrows} [\text{Active GEF·GDP-Rho}] \xrightarrow{kcat_{GEF}} [\text{Active GEF}] + [\text{GTP-Rho}]$<br>+ [GDI] ⇅ $Km_{GEF/GDI}$ (on [Active GEF]); + [GDI] ⇅ $Km_{GEF/GDI}$ (on [Active GEF·GDP-Rho])<br>$[\text{GDI·Active GEF}] + [\text{GDP-Rho}] \overset{Km_{GEF/Rho}}{\rightleftarrows} [\text{GDI·Active GEF·GDP-Rho}]$	$\frac{d[\text{GTP-Rho}]}{dt} = \frac{kcat_{GEF}[\text{Active GEF}][\text{GDP-Rho}]}{Km_{GEF/Rho}(1 + \frac{[\text{GDI}]}{Km_{GEF/GDI}}) + [\text{GDP-Rho}](1 + \frac{[\text{GDI}]}{Km_{GEF/GDI}})}$	$Km_{GEF/Rho} = 24.5\ \mu M$<br>$kcat_{GEF} = 5.64\ min^{-1}$<br>$Km_{GEF/GDI} = 1\ \mu M$	[Zhang, 2000]<br>[Zhang, 2000]<br>arbitrary
re5	A, D	$[\text{GTP-Rho}] + [\text{GAP}] \overset{Km_{GAP/Rho}}{\rightleftarrows} [\text{GTP-Rho·GAP}] \xrightarrow{kcat_{GAP}} [\text{GDP-Rho}] + [\text{GAP}]$	$\frac{d[\text{GDP-Rho}]}{dt} = \frac{kcat_{GAP}[\text{GTP-Rho}][\text{GAP}]}{Km_{GAP/Rho} + [\text{GTP-Rho}]}$	$Km_{GAP/Rho} = 4.48\ \mu M$<br>$kcat_{GAP} = 95.9\ min^{-1}$	[Zhang, 2000]<br>[Zhang, 2000]
	B, C	$[\text{GAP}] + [\text{GTP-Rho}] \overset{Km_{GAP/Rho}}{\rightleftarrows} [\text{GAP·GTP-Rho}] \xrightarrow{kcat_{GAP}} [\text{GAP}] + [\text{GDP-Rho}]$<br>+ [GDI] ⇅ $Km_{GAP/GDI}$ (on [GAP]); + [GDI] ⇅ $Km_{GAP/GDI}$ (on [GAP·GTP-Rho])<br>$[\text{GDI·GAP}] + [\text{GTP-Rho}] \overset{Km_{GAP/Rho}}{\rightleftarrows} [\text{GDI·GAP·GTP-Rho}]$	$\frac{d[\text{GDP-Rho}]}{dt} = \frac{kcat_{GAP}[\text{GAP}][\text{GTP-Rho}]}{Km_{GAP/Rho}(1 + \frac{[\text{GDI}]}{Km_{GAP/GDI}}) + [\text{GTP-Rho}](1 + \frac{[\text{GDI}]}{Km_{GAP/GDI}})}$	$Km_{GAP/Rho} = 4.48\ \mu M$<br>$kcat_{GAP} = 95.9\ min^{-1}$<br>$Km_{GAP/GDI} = 0.1\ \mu M$	[Zhang, 2000]<br>[Zhang, 2000]<br>arbitrary
re6	A, B, C, D	$[\text{GDP-Rho}] + [\text{GDI}] \underset{k_5}{\overset{k_4}{\rightleftarrows}} [\text{GDP-Rho·GDI}]$	$\frac{d[\text{GDP-Rho·GDI}]}{dt} = k_4[\text{GDP-Rho}][\text{GDI}] - k_5[\text{GDP-Rho·GDI}]$	$k_4 = 0.5\ \mu M^{-1} min^{-1}$<br>$k_5 = 0.05\ min^{-1}$	[Lipshtat, 2010]<br>[Lipshtat, 2010]
re7	A, B, C, D	$[\text{GTP-Rho}] + [\text{GDI}] \underset{k_7}{\overset{k_6}{\rightleftarrows}} [\text{GTP-Rho·GDI}]$	$\frac{d[\text{GTP-Rho·GDI}]}{dt} = k_6[\text{GTP-Rho}][\text{GDI}] - k_7[\text{GTP-Rho·GDI}]$	$k_6 = 0.5\ \mu M^{-1} min^{-1}$<br>$k_7 = 0.05\ min^{-1}$	[Lipshtat, 2010]<br>[Lipshtat, 2010]
re8	A, B, C, D	$[\text{GTP-Rho}] + [\text{Effector}] \underset{k_9}{\overset{k_8}{\rightleftarrows}} [\text{GTP-Rho·Effector}]$	$\frac{d[\text{GTP-Rho·Effector}]}{dt} = k_8[\text{GTP-Rho}][\text{ Effector}] - k_9[\text{GTP-Rho·Effector}]$	$k_8 = 28.2\ \mu M^{-1} min^{-1}$<br>$k_9 = 0.18\ min^{-1}$	[Rose, 2005]<br>[Rose, 2005]

Zhang B, Zhang Y, Wang Z, Zheng Y (2000) J Biol Chem 275: 25299-25307.

Lipshtat A, Jayaraman G, He JC, Iyengar R (2010) Proceedings of the National Academy of Sciences 107: 1247-1252.

Rose R, Weyand M, Lammers M, Ishizaki T, Ahmadian MR, Wittinghofer A (2005) Nature 435: 513-518.

* corresponding to the reaction numbers in Figure 1.

** corresponding to the models in Figure 1.
